# QuantiFERON-TB Gold In-Tube Assay for Screening Arthritis Patients for Latent Tuberculosis Infection before Starting Anti-Tumor Necrosis Factor Treatment

**DOI:** 10.1371/journal.pone.0119260

**Published:** 2015-03-06

**Authors:** Hyun Lee, Hye Yun Park, Kyeongman Jeon, Byeong-Ho Jeong, Ji-Won Hwang, Jaejoon Lee, Hoon-Suk Cha, Eun-Mi Koh, Eun-Suk Kang, Won-Jung Koh

**Affiliations:** 1 Division of Pulmonary and Critical Care Medicine, Department of Medicine, Samsung Medical Center, Sungkyunkwan University School of Medicine, Seoul, Korea; 2 Division of Rheumatology, Department of Medicine, Samsung Medical Center, Sungkyunkwan University School of Medicine, Seoul, Korea; 3 Department of Laboratory Medicine and Genetics, Samsung Medical Center, Sungkyunkwan University School of Medicine, Seoul, Korea; Hopital Raymond Poincare - Universite Versailles St. Quentin, FRANCE

## Abstract

**Background:**

Patients undergoing anti-tumor necrosis factor (TNF) treatment are at an increased risk of reactivating a latent tuberculosis infection (LTBI). This study evaluated the effectiveness of the QuantiFERON-TB Gold In-Tube (QFT) assay for diagnosing LTBI in arthritis patients undergoing anti-TNF treatment.

**Methods:**

We enrolled 342 consecutive patients from August 2007 to October 2013: 176 (51.5%) patients with ankylosing spondylitis and 166 (48.5%) with rheumatoid arthritis. Screening tests included tuberculin skin test (TST) and QFT assay. Positive QFT results, regardless of TST results, were considered an indicator for LTBI treatment.

**Results:**

Bacillus Calmette-Guérin scars were found in 236 (69.0%) patients. Of 342 patients, TST and QFT were positive in 122 (35.7%) and 103 (30.1%) patients, respectively, and discordant in 101 (29.5%) patients. During a median follow-up duration of 41.7 months, five patients (1.5%) developed TB in a median of 20.8 months after initiation of anti-TNF treatment (428/100,000 person-years). TB did not occur in 62 TST+/QFT+ patients who received LTBI treatment. Of 41 TST−/QFT+ patients who received LTBI treatment, one (2.4%) developed TB 20.5 months after starting anti-TNF treatment (705/100,000 person-years). Of 60 TST+/QFT− patients who did not receive LTBI treatment, two (3.3%) developed TB 20.8 and 22.0 months after starting anti-TNF treatment (871/100,000 person-years). Of 179 TST−/QFT− patients, two (1.1%) developed TB 7.2 and 22.7 months, respectively, after initiating anti-TNF treatment (341/100,000 person-years). TB incidence rate during the follow-up period did not differ among TST−/QFT+, TST+/QFT−, and TST−/QFT− patients (P = 0.661).

**Conclusion:**

QFT might be used instead of TST for diagnosing LTBI in patients before starting anti-TNF therapy in countries, such as Korea, where the TB prevalence is intermediate and the BCG vaccination is mandatory at birth. In the absence of a true gold standard test for LTBI, however, there is still a risk of TB development during anti-TNF treatment.

## Introduction

The introduction of biological agents such as anti-tumor necrosis factor (TNF)-α, has had a profound effect on the management of rheumatic arthritis, including both rheumatoid arthritis (RA) and ankylosing spondylitis (AS) [1, 2]. However, TNF-α is also a key cytokine in host defense against intracellular infections, such as *Mycobacterium tuberculosis* infection. Because of the risk of developing active tuberculosis (TB) with use of TNF-α antagonists [3, 4], patients should be screened for latent tuberculosis infections (LTBI) before starting anti-TNF treatment [5, 6].

Previously, many guidelines for the diagnosis of LTBI have relied on the tuberculin skin test (TST), despite its limitations [7–10]. The TST may produce false-positive results owing to prior Bacillus Calmette-Guérin (BCG) vaccination or nontuberculous mycobacterial infection; this poor specificity can lead to unnecessary LTBI treatment, with the risk of drug toxicity [11, 12]. Moreover, either the inflammatory disorder itself or the immunosuppressive treatment may lead to false-negative TST results [13].

Recently, whole-blood interferon-γ release assays (IGRAs), such as the QuantiFERON-TB Gold In-Tube (QFT; Cellestis, Carnegie, VIC, Australia) and the T-SPOT.TB assay (Oxford Immunotec, Abingdon, United Kingdom), were introduced for the diagnosis of LTBI [14]. In many studies comparing IGRA and TST, IGRA has been found to be more specific, better correlated with the degree of tuberculosis exposure, and less affected by prior BCG vaccination [15]. In addition, because the immunosuppressive treatment has a weaker effect on the IGRA, prior studies have suggested that IGRA is more effective than TST for LTBI screening in immune-mediated inflammatory diseases, including RA [16–18]. Some current national guidelines for screening prior to anti-TNF treatment recommend the use of the IGRA instead of the TST [19, 20].

Nevertheless, it is currently unclear whether the IGRA is superior to the TST or whether the IGRA can be used in arthritis patients as an alternative to the TST, and the exact screening approach and algorithm remain controversial [14, 21]. Some studies have suggested that a dual testing strategy including both TST and IGRA may be more accurate for the detection of LTBI before anti-TNF treatment than IGRA alone [22–24].

In a previous study, we reported a comparison of TST and the QFT assay for LTBI screening in 107 Korean arthritis patients before initiating anti-TNF treatment [25]. In that study, no patients developed active TB during a median of 18 months of anti-TNF treatment, including the 16 patients who tested positive by TST but negative by QFT and who were not treated for LTBI [25]. In the present study, we re-evaluated the usefulness of the QFT assay for diagnosis of LTBI in arthritis patients who received anti-TNF treatment in Korea, where the incidence of TB is intermediate (70–90/100,000 per year) and BCG vaccination is mandatory at birth [26]. This study enrolled 342 patients, including 107 patients from our previous study, and reported the long-term follow-up data.

## Patients and Methods

### Patients

**Fig 1:**
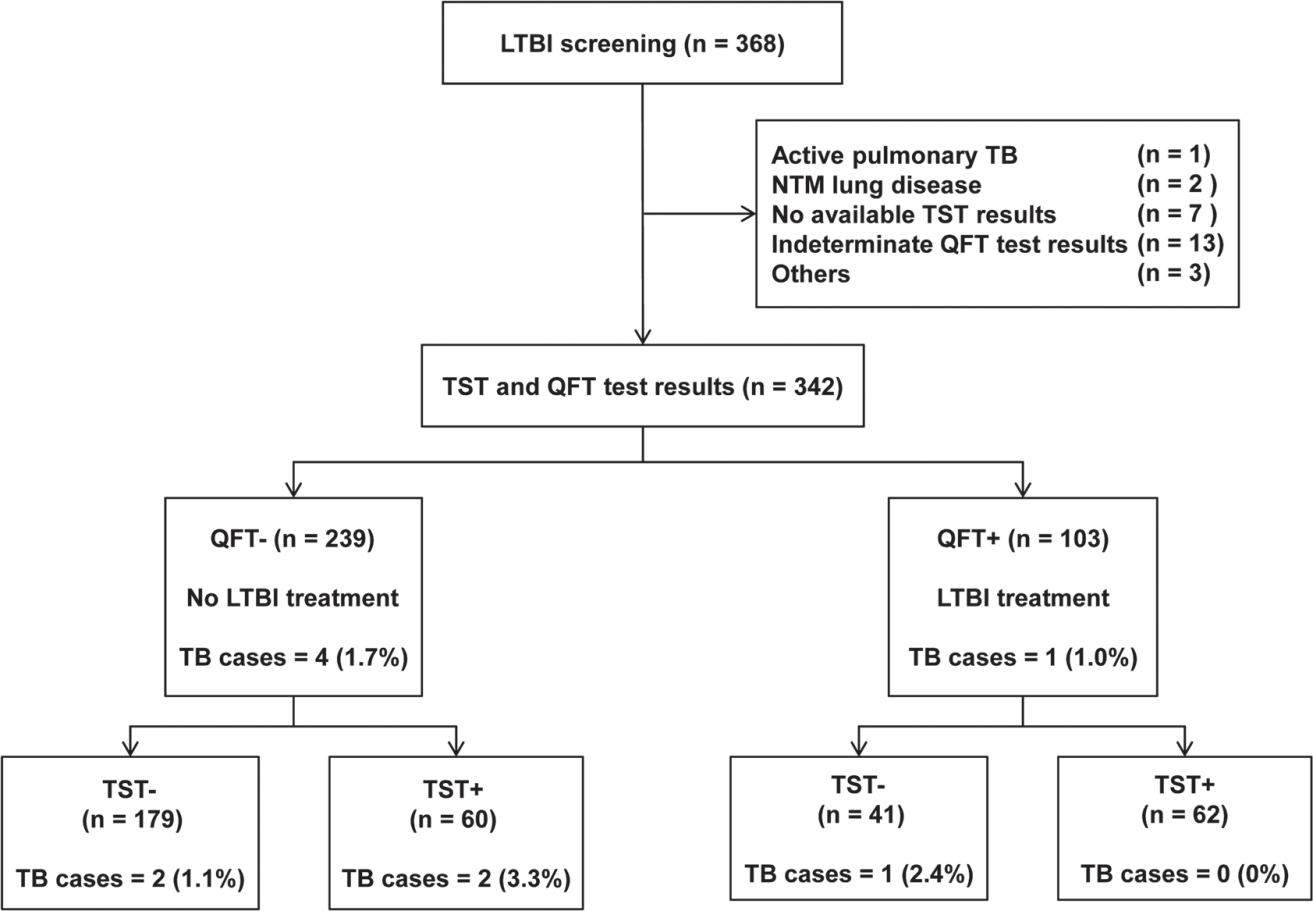
Flow diagram of the study population. LTBI = latent tuberculosis infection; TB = tuberculosis; NTM = nontuberculous mycobacteria; TST = tuberculin skin test; QFT = QuantiFERON-TB Gold In-Tube.

We evaluated the medical records of 368 consecutive patients with inflammatory arthritis, including those with RA and AS, who visited Samsung Medical Center (a 1961-bed referral hospital in Seoul, South Korea) between August 2007 to October 2013 to evaluate LTBI before starting anti-TNF treatment. RA and AS were diagnosed based on the proposed criteria [27, 28]. Cases were excluded if patients had (1) active pulmonary TB (n = 1), (2) nontuberculous mycobacterial lung disease (n = 2), (3) no available TST results (n = 7), (4) indeterminate QFT test results (n = 13), or (5) others such as a documented TST conversion (n = 3). The final analysis was performed on 342 patients (Fig. 1).

The Institutional Review Board of Samsung Medical Center approved this study and has given permission for it to be reviewed and published, including information obtained from patient records (IRB No. 2014-08-014). Informed consent was waived because of the retrospective nature of the study, and patient information was anonymized and de-identified prior to analysis.

### Screening, diagnosis and treatment of LTBI

LTBI was diagnosed from medical histories and screening tests. The evaluation of medical history included current symptoms, history of TB treatment, and recent history of contact with a patient with active pulmonary TB. Both the TST and the QFT assay were performed on the same day as screening tests for LTBI. The QFT assay was performed in accordance with the manufacturer’s instructions; the test was interpreted as positive when the result was ≥ 0.35 IU/mL. The TST was performed on the volar side of the forearm using the Mantoux method with two tuberculin units of purified protein derivative RT23 (Statens Serum Institut; Copenhagen, Denmark) [25]. A positive test was defined as an induration ≥ 10 mm after 48–72 hours according to the Korean National Guidelines [29, 30].

LTBI treatment was administered as necessary based on the QFT assay results, regardless of the TST results. Isoniazid for 9 months, rifampin for 4 months, or both isoniazid and rifampin for 3 months were used as the LTBI treatment regimen, selected at the discretion of the treating physician. Anti-TNF treatment was initiated after at least 4 weeks of LTBI treatment [5, 31].

### Statistical analysis

Continuous variables are presented as medians and interquartile range (IQR) and compared using a Student’s *t*-test or a Mann-Whitney *U* test, as appropriate. Categorical variables are presented as numbers (percentages) and compared using the Pearson χ^2^ test or Fisher’s exact test. The concordance between TST and QFT test results was assessed by means of kappa (κ) statistics with a κ-value > 0.75 representing excellent agreement, 0.40 to 0.75 representing fair to good agreement, and < 0.40 representing poor agreement. TB incidence over time was calculated using the Gehan-Breslow-Wilcoxon test in univariate analysis and a Cox regression model in a multivariate analysis including age, sex and factors with P-values < 0.20 in univariate analyses. All tests were two-sided, and a P-value < 0.05 was considered significant. All statistical analyses were performed using SPSS 21.0 (SPSS Inc., Chicago, IL, USA).

## Results

### Patient characteristics

Of 342 patients, 190 (55.6%) were male, and the median age was 40.0 years (IQR, 30.0–53.0 years). BCG scars were found in 236 patients (69.0%), and 23 patients (6.7%) had a history of TB treatment. The diagnosis of RA was established in 166 patients (48.5%) and AS in 176 patients (51.5%). Immunosuppressive drugs were administered to 194 patients (56.7%), and drugs included glucocorticoids (n = 163, 47.7%), methotrexate (n = 161, 47.1%) and leflunomide (n = 54, 15.8%). Some patients received more than two types of immunosuppressive drugs.

The clinical characteristics of the patients with RA and AS differed. Patients with AS were more likely to be male (159/176, 90.3%), whereas a majority of the patients with RA were female (135/166, 81.3%; P < 0.001). The patients with AS were younger (median age, 33 years) than those with RA (median age, 50.5 years; P < 0.001). Patients with AS were more likely to have a BCG scar (136/176, 77.3%) compared with those with RA (100/166, 60.2%; P = 0.001). The percentage of RA patients receiving immunosuppressive drugs was higher than that of patients with AS (P < 0.001; Table 1).

**Table 1:** Baseline characteristics of patients with rheumatoid arthritis or ankylosing spondylitis.

	Total (n = 342)	RA (n = 166)	AS (n = 176)	P value
Age, years	40.0 (30.0–53.0)	50.5 (40.0–62.0)	33.0 (27.0–40.0)	< 0.001
Sex, male	190 (55.6)	31 (18.7)	159 (90.3)	< 0.001
History of TB treatment	23 (6.7)	13 (7.8)	10 (5.7)	0.428
BCG scar	236 (69.0)	100 (60.2)	136 (77.3)	0.001
Immunosuppressive drugs				< 0.001
Glucocorticoid	163 (47.7)	137 (82.5)	26 (14.8)	
Methotrexate	161 (47.1)	145 (87.3)	16 (9.1)	
Leflunomide	54 (15.8)	53 (31.9)	1 (0.6)	
None	148 (43.3)	2 (1.2)	146 (83.0)	
TST-positive	122 (35.7)	38 (22.9)	84 (47.7)	< 0.001
QFT-positive	103 (30.1)	52 (31.3)	51 (29.0)	0.636

The data are presented as median and interquartile range or as number (%).

RA = rheumatoid arthritis; AS = ankylosing spondylitis; BCG = Bacillus Calmette-Guérin; TB = tuberculosis; TST = tuberculin skin test; QFT = QuantiFERON-TB Gold In-Tube.

### Diagnosis and treatment of LTBI

Overall, the TST was positive in 122 patients (35.7%) and the QFT assay was positive in 103 patients (30.1%). Sixty-two patients (18.1%) showed positive results for both TST and QFT assay (TST+/QFT+), and 179 (52.3%) showed negative results for both TST and QFT assay (TST−/QFT−). Of 101 patients with discordance between the TST and QFT assay results, 60 (17.5%) had TST-positive and QFT-negative results (TST+/QFT−), and 41 (12.0%) had TST-negative and QFT-positive results (TST−/QFT+) (Fig. 1). The clinical characteristics of patients who had discordant results on the TST and QFT assay are presented in Table 2. The TST+/QFT− patients were typically younger, male and more likely to have a BCG scar and AS. In contrast, the TST−/QFT+ patients tended to be older, female, more likely to have RA and more likely to receive immunosuppressive drugs.

**Table 2:** Characteristics of patients with discordant results on the TST and QFT assays.

	TST+/QFT− (n = 60)	TST−/QFT+ (n = 41)	P value
Age, years	33.0 (27.0–42.8)	53.0 (40.5–60.0)	< 0.001
Sex, male	48 (80.0)	21 (51.2)	0.002
History of TB treatment	5 (8.3)	3 (7.3)	>0.999
BCG scar	49 (81.7)	24 (58.5)	0.011
Underlying disease			< 0.001
Rheumatoid arthritis	10 (16.7)	24 (58.5)	
Ankylosing spondylitis	50 (83.3)	17 (41.5)	
Immunosuppressive drugs	18 (30.0)	28 (68.3)	<0.001

The data are presented as median and interquartile range or as number (%).

TST = tuberculin skin test; QFT = QuantiFERON-TB Gold In-Tube; TB = tuberculosis; BCG = Bacillus Calmette-Guérin.

The kappa coefficient value for the agreement between TST and QFT tests was poor (κ = 0.333, 95% confidence interval [CI]: 0.229–0.437, P <0.001). The agreement between TST and QFT assay in AS patients was low (κ = 0.224, 95% CI: 0.091–0.357, P = 0.001), whereas the agreement in RA patients was moderate (κ = 0.486, 95% CI: 0.341–0.631, P < 0.001; Table 3).

**Table 3:** Agreement between TST and QFT-IT in patients with rheumatoid arthritis or ankylosing spondylitis.

	Rheumatoid arthritis (n = 166)			Ankylosing spondylitis (n = 176)		
	TST-negative	TST-positive	Total	TST-negative	TST-positive	Total
QFT-negative	104	10	114	75	50	125
QFT-positive	24	28	52	17	34	51
Total	128	38	166	92	84	176
Kappa value	κ = 0.486, (95% CI: 0.341–0.631, P < 0.001)			κ = 0.224, (95% CI: 0.091–0.357, P = 0.001)		

The data are presented as numbers.

TST = tuberculin skin test; QFT = QuantiFERON-TB Gold In-Tube.

During the study period, LTBI treatment was indicated in 103 patients (30.1%) before anti-TNF treatment, and all of them completed LTBI treatment. Of 103 patients who were diagnosed with LTBI, 85 (82.5%) received isoniazid and rifampin for 3 months, and regimens were changed to isoniazid or rifampin due to side effects in five patients. Fourteen patients (13.6%) received rifampin for 4 months, and four (3.9%) received isoniazid for 9 months.

### Follow-up after initiation of TNF antagonist

Of TNF-α antagonists, etanercept, adalimumab and infliximab were administered in 183 (53.5%), 176 (51.5%) and 54 (15.8%) patients, respectively. Some patients switched from one TNF-α antagonist to another during treatment.

During a median of 41.7 months (IQR, 21.5–59.1 months) of follow-up period, five patients (1.5%) developed active TB in a median of 20.8 months (IQR, 13.9–22.4 months) after initiation of anti-TNF treatment (428/100,000 person-years). Among them, one patient had pulmonary TB, two patients had both pulmonary and extra-pulmonary TB, and two patients had extra-pulmonary TB. Pulmonary TB was confirmed by sputum or a bronchial washing culture. TB pleurisy and TB peritonitis were diagnosed based on clinical, radiographic and laboratory findings (lymphocyte-dominant exudate with elevated level of adenosine deaminase > 60 IU/L in body fluid) (Table 4).

**Table 4:** Characteristics of five patients who developed active tuberculosis after initiation of anti-TNF therapy.

	Patient 1	Patient 2	Patient 3	Patient 4	Patient 5
Age	62	51	41	27	33
Sex	Female	Female	Male	Male	Male
Underlying disease	RA	RA	AS	AS	AS
History of TB treatment	No	No	No	No	No
BCG scar	Yes	Yes	Yes	No	Yes
Baseline chest radiography	Normal	Fibrotic nodules^1^	Normal	Normal	Normal
Baseline TST (induration, mm)	Negative (0)	Negative (7)	Negative (6)	Positive (10)	Positive (10)
Baseline QFT (IU/mL)	Negative (0.01)	Negative (0.33)	Positive (0.58)	Negative (-0.01)	Negative (0.08)
LTBI treatment	No	No	Yes	No	No
TNF antagonists	Adalimumab	Infliximab	Etanercept followed by Infliximab	Adalimumab	Infliximab
Time to diagnosis of TB after start of TNF antagonists, months	22.7	7.2	20.5	22.0	20.8
Sites of TB	TB pleurisy	Pulmonary TB & TB pleurisy	Pulmonary TB & TB peritonitis	TB peritonitis	Pulmonary TB
Culture positivity	No	Yes	Yes	No	Yes
Drug susceptibility	NA	Sensitive	MDR	NA	Sensitive

RA = rheumatoid arthritis; AS = ankylosing spondylitis; TB = tuberculosis; BCG = Bacillus Calmette-Guérin; TST = tuberculin skin test; QFT = QuantiFERON-TB Gold In Tube; LTBI = latent tuberculosis infection; TNF = tumor necrosis factor; NA = not available; MDR = multidrug-resistant.

^1^Retrospective review of baseline chest radiography showed several tiny fibrotic nodules in the right upper lobe. This lesion was not recognized for indication for treatment of latent tuberculosis infection by the attending physician at that time.

Of 179 patients who had TST−/QFT− results and did not receive LTBI treatment, active TB developed in two (1.1%) 7.2 and 22.7 months after initiating TNF-α antagonist treatment, respectively (341/100,000 person-years). Of 60 patients who had TST+/QFT− results and did not receive LTBI treatment, active TB developed in two (3.3%) 20.8 and 22.0 months after starting anti-TNF treatment, respectively (871/100,000 person-years). Of 41 patients who had TST−/QFT+ results and received LTBI treatment, pulmonary TB and TB peritonitis developed in one (2.4%) 20.5 months after starting anti-TNF treatment (705/100,000 person-years), whereas active TB did not develop in 62 patients who had TST+/QFT+ results and received LTBI treatment during a median of 41.5 months (IQR, 20.8–59.1 months) follow-up period.

**Fig 2:**
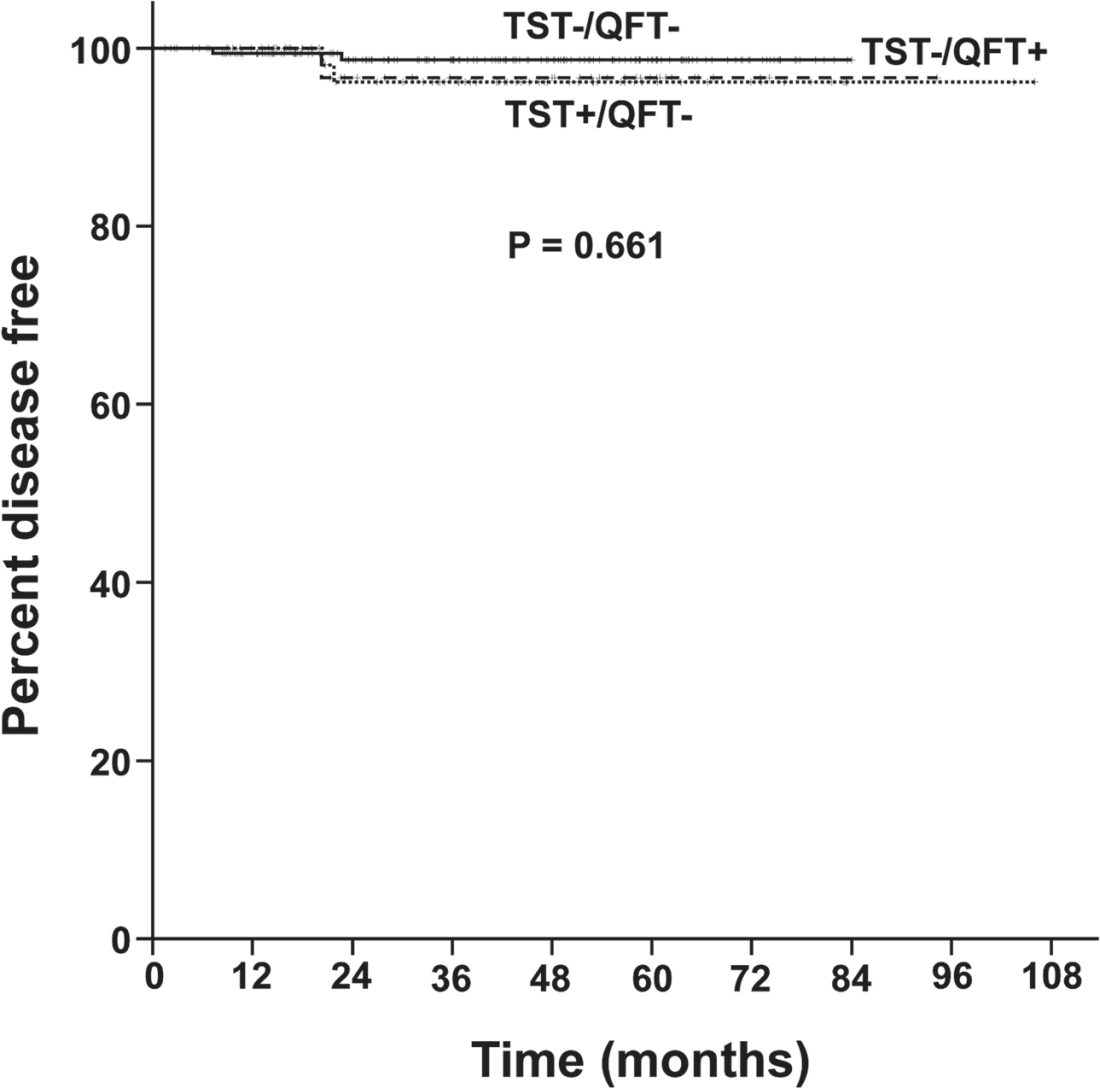
The proportion of tuberculosis-free patients with TST+/QFT− (dotted line), TST−/QFT+ (dashed line) and TST−/QFT− results (solid line) in Kaplan-Meier curves. QFT = QuantiFERON-TB Gold In-Tube; TST = tuberculin skin test.

The median follow-up durations after initiation of anti-TNF treatment was 38.5 months (IQR 19.2–58.6 months) in the TST−/QFT− patients, 44.8 months (IQR 32.8–58.6 months) in the TST+/QFT− patients and 48.3 months (IQR 19.6–60.8 months) in the TST−/QFT+ patients. TB incidence rate during the follow-up period was not different among TST−/QFT− patients, TST+/QFT− patients and TST−/QFT+ patients (P = 0.661, Fig. 2), which remained consistent even after adjustments were made for age, sex, BCG scars, TB treatment history, underlying diseases and use of immunosuppressant drugs (P = 0.673).

## Discussion

In the present study, approximately 30% of patients with RA or AS were diagnosed with LTBI using the IGRA and received LTBI treatment. During a 41.7-month median follow-up period, five patients (1.5%) developed active TB in a median of 20.8 months after initiation of anti-TNF treatment. The incidence rate of TB during the follow-up period did not differ between TST+/QFT− patients and TST−/QFT− patients, although all patients did not receive LTBI treatment. This study suggested that IGRA could be used instead of TST for diagnosing LTBI in patients before starting anti-TNF treatment in countries such as Korea, where the TB prevalence is intermediate and the BCG vaccination is mandatory at birth.

Investigation of LTBI in arthritis patients who received anti-TNF treatment is very important because such patients are at increased risk of LTBI reactivation [3–5]. To prevent reactivation of LTBI, a number of countries have generated national guidelines to address LTBI before initiation of anti-TNF therapy. However, LTBI screening with TST has several drawbacks, such as a potential for false-positive results in individuals who have receive a BCG vaccination, particularly given after infancy [32] and a potential for false-negative results in individuals with impaired cellular immunity [11–13].

The overall agreement between TST and QFT assay in the present study was poor (κ = 0.333), similar to the results of our previous study [25]. We found comparable levels of positive test results with the QFT assay, but a significant difference in TST results between RA and AS patients. This discrepancy may be explained by the limitations of the TST. Immunosuppressive drugs are associated with anergry of TST [33], and RA itself is well known to suppress TST response [13]. In the present study, in addition to RA disease itself, immunosuppressive drugs were more commonly used in RA patients compared with AS patients. In addition, this effect might be greater in AS patients with mostly young patients than in RA patients, because false-positive effect of BCG vaccination on TST wanes with ages [34]. These results suggest that a substantial proportion of AS patients without LTBI may be inaccurately diagnosed with LTBI by the TST, and a substantial proportion of RA patients with LTBI may be missed by the TST. Therefore, the QFT assay might prevent both the overtreatment of LTBI in AS patients with false-positive TST results and the underdiagnosis of LTBI in RA patients due to false-negative TST results [25].

Considering that the TST has a low specificity for LTBI diagnosis and its result is affected by many other factors, IGRA is regarded as an alternative to TST in the diagnosis of LTBI, especially in patients who have previously received a BCG vaccination [5, 31, 35]. Whether or not arthritis patients should be screened for LTBI using the IGRA instead of the TST, however, is still unclear and controversial [14]. To date, there have been few longitudinal studies that have evaluated the predictive role of IGRA in arthritis patients before anti-TNF therapy [11]. In the present study, the diagnosis and treatment of LTBI relied on the QFT assay rather than the TST, and 60 patients with TST+/QFT− did not receive LTBI treatment. Although two patients developed active TB during a median of 44.8 months of anti-TNF therapy in these patients, the incidence of active TB during the follow-up duration was not different between TST+/QFT− patients and TST−/QFT− patients. These findings are consistent with the recently published report from France, in which prior BCG vaccination was also frequent [11]. In that study, none of the 50 patients with positive TST results at a 10 mm cut-off point and negative IGRA results developed TB during the 12 months of follow-up, although they received anti-TNF therapy without LTBI treatment [11]. In addition, there were no statistically significant differences in the development of active TB between the subgroups when a cut off value of 5mm was used.

Some experts recommend a dual testing strategy (or ‘either test positive’ strategy) in which both TST and IGRA are used and either positive be accepted as indication of LTBI [21, 36–38]. Several previous studies suggested that IGRA alone, without TST, might not sufficiently identify all patients at risk due to false-negative screening results, particularly in immunosuppressive individuals [22–24]. The use of both TST and IGRA may maximize sensitivity for detecting LTBI but may also reduce specificity. Particularly in areas of BCG vaccination use, some patients with discordant TST-positive and IGRA-negative results due to BCG exposure will be wrongly diagnosed with LTBI, and this strategy could result in increased cost, possible adverse effects in response to LTBI treatment and delay in anti-TNF therapy [11].

Current Korean guidelines recommend that 1) TST alone, 2) IGRA alone or 3) both TST and IGRA be used as necessary for LTBI screening in immunocompromised subjects [29, 30]. A recent study undertaken in South Korea reported that an ‘either test positive’ strategy was useful for LTBI diagnosis before anti-TNF treatment in patients with immune-mediated inflammatory diseases [24]. In that study, LTBI was diagnosed in 46.0% (198/430) of patients based on TST or T.SPOT assay, and 0.9% (4/430) of patients developed active TB during the median follow-up duration of 29.1 months (382/100,000 person-year) [24, 39]. The figures were very similar to the results in the present study, as 1.5% (5/342) of patients developed active TB during a median follow-up duration of 41.7 months (428/100,000 person-year), and only 31.0% (103/342) of patients were diagnosed with LTBI based on QFT assay. Our study supports the use of IGRA instead of TST to result in fewer arthritis patients with an indication for anti-TNF therapy and treated for LTBI without increasing their risk of developing active TB. These data support recent expert recommendations suggesting that IGRAs are superior to the TST in identifying individuals with a history of BCG vaccination who are at risk of developing TB during TNF-α antagonist therapy [5].

In the present study, one patient with LTBI treatment developed multidrug-resistant TB, which could not be prevented by LTBI treatment with isoniazid or rifampin. Other four patients developed active TB in a median of 21.4 months (IQR, 10.6–22.5 months) after the initiation of anti-TNF therapy, including two TST+QFT− patients who developed TB 22.0 or 20.8 months after anti-TNF therapy. Considering that the risk of developing TB due to the reactivation of LTBI was highest during the first 6–12 months of anti-TNF therapy [3, 40–42], there was a possibility that some of these patients could develop TB due to recent new infection with *M*. *tuberculosis*. The role of repeated or periodic screening after initiating anti-TNF therapy requires further evaluation [31, 43, 44].

Previous studies suggested that the risk of TB is higher for patients receiving anti-TNF-α antibodies, such as infliximab or adalimumab, than it is for those receiving the soluble TNF-α receptor, etanercept. In addition, the median time of TB onset after receiving monoclonal antibody therapy was shorter than that of the soluble receptor [45–49]. In our study, all five patients who developed active TB received infliximab or adalimumab. These findings suggest that patients receiving monoclonal anti-TNF-α antibodies should be monitored more closely than those receiving the soluble TNF-α receptor.

Our study had several limitations. First, the present study was conducted at a single referral center in an area with an intermediate TB burden. Therefore, it may not be appropriate to apply the results of our study to patients in areas with low TB incidence and low prevalence of BCG vaccinations. Second, we used only one of the two available IGRAs, *i*.*e*., the QFT assay. Therefore, our results cannot be used to predict the results of all IGRAs. Third, there was a trend for more TB cases among TST+/QFT− patients compared with TST−/QFT− patients, although the incidence rates were not statistically different. Therefore, the number of patients who actually developed TB may be insufficient to detect the difference between the IGRA-only strategy and the ‘either test positive’ strategy, although we enrolled a large number of patients.

In conclusion, IGRA might be used instead of TST for diagnosing LTBI in patients before starting anti-TNF therapy in countries such as Korea, where the TB prevalence is intermediate and the BCG vaccination is mandatory at birth. In the absence of a true gold standard test for LTBI, however, there is still a risk of developing TB during anti-TNF treatment.
